# Changes in Distribution of Dental Biofilm after Insertion of Fixed Orthodontic Appliances

**DOI:** 10.3390/jcm10235638

**Published:** 2021-11-29

**Authors:** Urszula Kozak, Agnieszka Lasota, Renata Chałas

**Affiliations:** 1 Chair and Department of Jaw Orthopaedics, Medical University of Lublin, 20-093 Lublin, Poland; agnieszkalasota@umlub.pl; 2 Department of Oral Medicine, Medical University of Lublin, 20-093 Lublin, Poland; renata.chalas@umlub.pl

**Keywords:** biofilm, brackets, caries, dental plaque, oral health, fixed orthodontic appliances

## Abstract

Good oral hygiene is an important factor in oral and general health, especially in orthodontic patients, because fixed appliances might impede effective oral hygiene and thus increase the risks of tooth decay, periodontal disease and general health complications. This study investigated the impact of fixed orthodontic appliances on the distribution of dental biofilm in teenagers. Supragingival plaque was assessed at T0, T1 and T2. The distribution of the biofilm was analyzed. Approximal Plaque Index (API) and Bonded Bracket Index (BBI) were used to measure the presence of dental plaque. After insertion of the fixed appliance, the dental plaque indices values in the orthodontically treated group were significantly higher (*p* < 0.05) than in the control group. Fixed orthodontic appliances caused significant changes in the distribution of the biofilm. This was characterized by the change of location of the dental plaque. In the orthodontic group, we observed an increase in the amount of the supragingival plaque on the vestibular surface of the teeth.

## 1. Introduction

Good oral hygiene is an important factor in oral health, especially in orthodontic patients, because fixed appliances might impede effective oral hygiene and thus increase the risks of periodontal problems, tooth caries and general health complications [1,2,3]. Dental plaque is a polymicrobial biofilm composed of various bacterial complexes, including commensal, symbiotic and potentially pathogenic species [4,5,6,7,8,9,10].

The microbiome consists of over 600 species that live in ecological niches of the oral cavity [11,12].

Clinical studies have assessed the role of the plaque in the initiation and progression of dental caries, gingival problems, periodontal disease and general health complications.

Many endogenous and exogenous factors, such as microorganisms and the patient’s immunological response, cause the loss of homeostasis of the oral microbiota and contribute to the development of pathological changes [13,14,15].

Fixed orthodontic appliances alter the oral microbiota during treatment. After insertion of the fixed appliance, an increase in the microbial population, particularly *Streptococcus* and *Lactobacillus*, was observed [16,17]. Orthodontic appliances may also influence the subgingival microbiota. Supragingival Gram-positive oral microbiota, especially *Streptococci* and *Actinomyces*, are the basic etiological factors of periodontal diseases, whereas, in gingivitis, an increase in the amount of Gram-negative bacteria (*Fusobacterium* and *Bacteroides*) is observed [18,19].

Literature indicates that after removal of the appliance, opportunistic bacteria, responsible for the development of severe diseases, were detected in the blood of orthodontic patients [13,14,15].

Measurement of dental biofilm is therefore fundamental in the evaluation of the orthodontic patients’ oral hygiene and in clinical studies assessing dental plaque.

Studies examining dental plaque accumulation indicate that most patients have a repeatable pattern of plaque accumulation. Some areas have low and others high amounts of dental plaque [20,21,22,23].

Assessment of plaque formation in patients without orthodontic appliances revealed that dental biofilm accumulated mainly on the approximal surfaces [24]. It is worth noting that oral hygiene on the vestibular surfaces with orthodontic brackets seems as difficult as in the interproximal region and requires accurate hygiene in these areas.

The methods used to assess plaque include planimetric analysis of plaque, which expresses the surface of the plaque as a percentage of the tooth surface covered with plaque, and the most used numerical categorical scale (index). Several such indices have been developed: Silness and Löe, Hein, Turesky, O’Leary and Quigley. The usefulness of these indicators for patients with braces should be questioned as visual judgments are generally based on the extent and thickness of the plaque near the gingival margin and the coronary expansion of the plaque. These indices are intended to reflect the typical model of development in plaque accumulation. Therefore, with fixed appliances, the presence of adhesive attachments and arch wires should be taken into consideration when the pattern of plaque accumulation is assessed.

It has been confirmed that the presence of biofilm on the tooth surface is a predictor of the development of caries in children.

This study investigated the impact of fixed orthodontic appliances on the distribution of dental biofilm.

## 2. Materials and Methods

### 2.1. Eligibility Criteria

Inclusion criteria:

- Generally healthy patients (patients without any mental or physical disability, systemic diseases and craniofacial disorders)
- 12–18 years old (according to the literature, age is a risk factor for neglection of oral hygiene; among teenagers during fixed appliances therapy more supragingival dental plaque was observed)
- Full permanent dentition

Exclusion criteria:

- Previous orthodontic treatment and surgical procedures;
- Non-carious enamel lesions (non-carious lesion (surfaces with developmental defects such as enamel hypoplasia, fluorosis, tooth wear) was defined as a change in enamel opacity and surface structure that is not related to cariogenic biofilm);
- Interruption of the enamel continuity;
- Poor oral hygiene (API > 40 %);
- Patients with IOTN grade 1 and 5.

Adequately to the study group, the control group was formed.

### 2.2. Sample Characteristics

**Figure 1:**
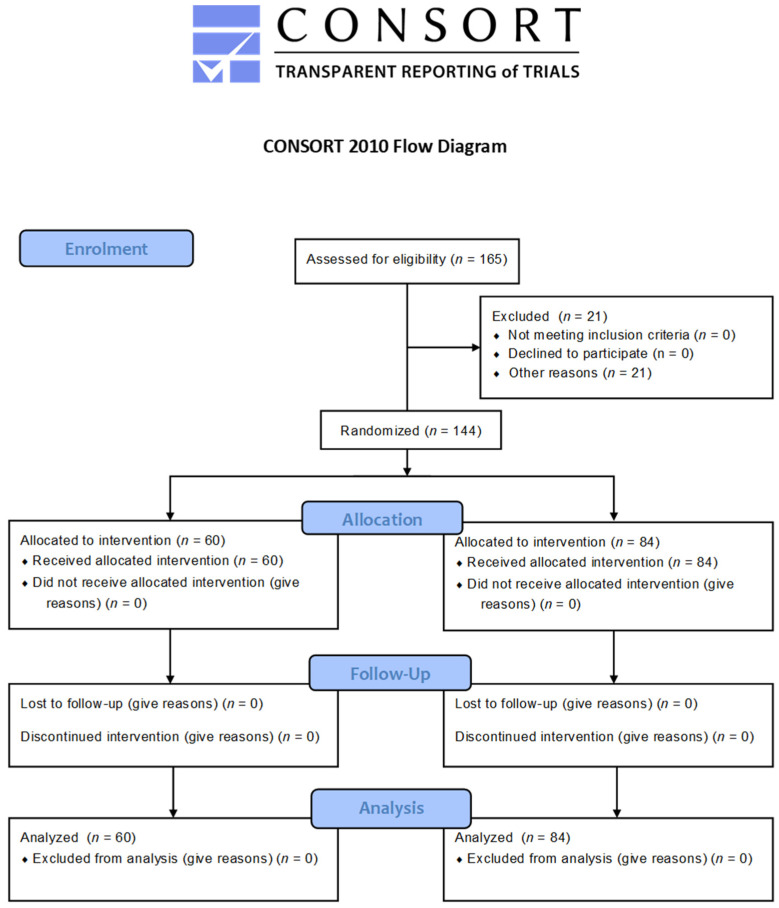
Flow diagram. A total of 144 participants completed the full study protocol.

The clinical-control observational study involved 144 patients; 94 girls and 50 boys participated in the study. Patients were divided into two groups (Figure 1):

- An orthodontically treated group including patients qualified for treatment with conventional fixed orthodontic appliances.
- A control group consisted of students attending schools located in the Lublin Province, Poland; in each of the schools, there were dental offices.

In total, 122 people aged 12–15 years (48 people in the orthodontic group and 74 in the control group) and 22 people aged 16–18 years (12 in the orthodontic group and 10 in the control group) were examined.

The mean age was 14.03 for the orthodontically treated group and 13.48 for the control group.

A total of 60 patients treated with fixed appliances (18 boys and 42 girls) and 84 control students attending schools (32 boys and 52 girls) were examined.

The majority of participants represented the urban environment (63.3% in the orthodontic group and 70.2% in the control group).

The study was approved by the Bioethical Committee of the Medical University of Lublin (no. KE-0254/169/2011), patients, parents/caregivers, school principals and dentists working for them.

### 2.3. Data Collection

Our study was divided into three stages:

I: An initial examination was carried out during a visit preceding the insertion of fixed appliance—T0;

II: Then one month after the placement of the fixed appliance—T1;

III: And finally after six months of treatment—T2.

Patients in orthodontic group were treated with conventional metal brackets.

A 12 NiTi wire was used at the beginning of the treatment. After 6 months of therapy, 18 SS wire was used.

The dental examination was carried out in accordance with WHO recommendations [25]. The physical examination was carried out in a dental office in the light of a shadeless lamp, using a flat mirror and periodontal probe WHO-621.

Orthodontic brackets and wires are placed on the vestibular surface of the teeth, thus impeding their self-cleaning with saliva, promoting retention of food remnants and deteriorating cleaning especially of the labial/buccal and interdental spaces. Because of these reasons, two indices were measured:

API (Approximal Plaque Index)—designed to assess the amount of plaque in the interdental spaces; used in this examination to compare the oral hygiene status between the two groups: orthodontically treated and control.

BBI (Bonded Bracket Index)—dedicated to patients with orthodontic braces. BBI was only used in orthodontically treated group to assess the oral hygiene status during the 6-month treatment period.

Measurements were performed by the same examiner, who received appropriate training and was calibrated by an experienced clinician to measure the presence of dental plaques with two API and BBI indices. Approximately 20% of the patients were re-examined to determine intraexaminer reliability, which was found to be 0.93.

### 2.4. Examination Sequence

Approximal Plaque Index (API) by Lange et al. [26]

**Figure 2:**
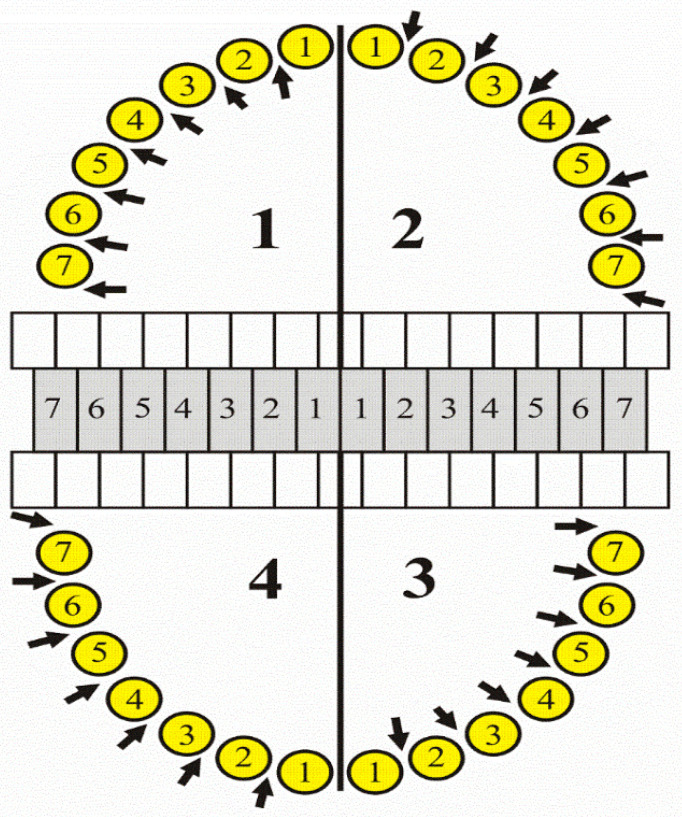
Diagram for determining Approximal Plaque Index (API). 1, 3—interproximal spaces are scored from the oral aspect; 2, 4—interproximal spaces are scored from the facial aspect.

The oral hygiene status was expressed numerically. The API was measured to assess the amount of uncleaned interdental space as a percentage (Figure 2).

The evaluation criterion was the presence (+) or absence (-) of dental plaque on the approximal areas. The API determines the percentage of the sum of the dental plaque surfaces in relation to the sum of all examined areas.

API values:

 < 25%, optimal oral hygiene

25–39%, good oral hygiene

40–69%, fair oral hygiene

70–100%, poor oral hygiene

Bonded Bracket Index (BBI) by Aloufi et al. [27]. 

**Figure 3:**
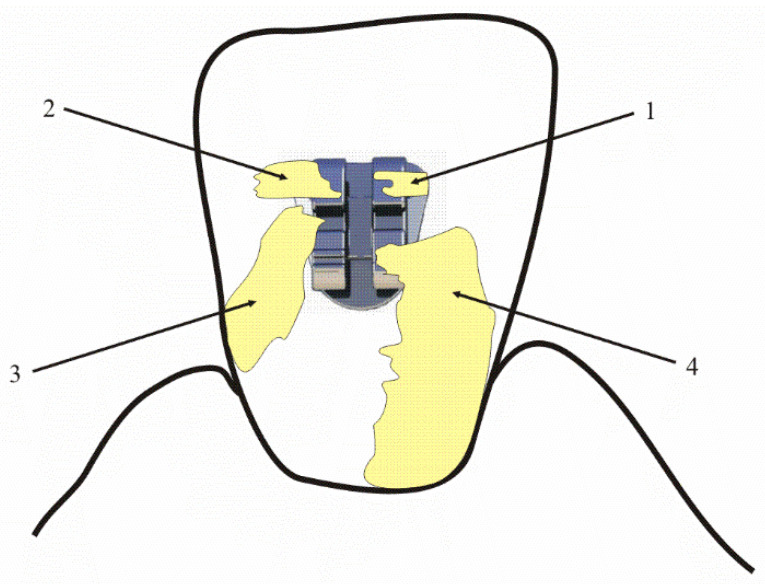
Schematic representation of the BBI: 1—plaque present on the bracket only; 2—plaque present on the bracket and the immediate adjacent tooth surface; 3—plaque present on the bracket and continuous to the interproximal surface; 4—a continuous layer of plaque extending from the bracket to the gingival margin.

Taking into account the difficulties in removing plaque from the area adjacent to the orthodontic bracket, Bonded Bracket Index was used in the study group to help monitor plaque biofilm control throughout the 6-month orthodontic treatment (Figure 3).

BBI aims to account for the effect of an orthodontic bracket on the distribution of dental plaque. While it refers to an orthodontic bracket, the categories emphasize spread toward and contact with the gingiva.

BBI grades:

1: Plaque present on the bracket only.

2: Plaque present on the bracket and the immediate adjacent tooth surface.

3: Plaque present on the bracket and continuous to the interproximal surface.

4: A continuous layer of plaque extending from the bracket to the gingival margin.

Orthodontically treated patients were informed that optimal oral hygiene is a prerequisite for starting the therapy. Extensive oral-hygiene instruction was given prior to the start of the trials. Patients from both groups were instructed to thoroughly clean teeth with a fluoride dentifrice after every meal and even after each snack, floss before brushing and rinse once a day with a sodium fluoride mouth rinse.

The health condition of the oral cavity was evaluated in the first months of treatment in order to verify the effectiveness of the conducted hygienic procedures and show patient sites that were particularly difficult to access. Intense instructions of oral-cavity hygiene status and frequently repeated motivation were given to patients from both groups.

### 2.5. Statistical Analysis

The results obtained in the clinical trial were statistically analyzed and are presented as descriptive analysis and U-test relations. The Mann–Whitney U test, ANOVA Friedman and Wilcoxon’s pairwise tests were performed for the Approximal Plaque Index and Wilcoxon’s pairwise test for the Bonded Bracket Index. A typical statistical-significance level of *p* = 0.05 was assumed for the entire analysis. The main calculations were performed and graphs were prepared in STATISTICA 8.0 PL (StatSoft, Poland).

## 3. Results

### Clinical Examination

Approximal Plaque Index (API) (Table 1, Table 2 and Table 3).

During the first study, the distribution of API values in the orthodontic treatment group and the control group did not differ significantly (*p* > 0.05). However, the distributions of API index values assessed in the second and third studies differed significantly (*p* < 0.05) in the orthodontic treatment group and the control group.

After insertion of the fixed appliance, the API values in the orthodontically treated group were significantly higher than in the control group, which indicates a deterioration of oral hygiene after insertion of the fixed appliance.

The lowest values of the API index were observed before the insertion of the braces, slightly higher values were recorded after a month of orthodontic therapy, while the highest values of the API index were observed after six months of treatment. In the control group, the best hygiene was observed in the second study, the API index was slightly higher in the first study, and the highest API values were obtained in the third study.

Bonded Bracket Index (BBI) (Table 4 and Table 5).

Comparison of BBI values assessed after one month and six months after the insertion of the fixed braces in the orthodontic treatment group differed significantly (*p* < 0.05). In the orthodontic group, the BBI values obtained one month after the insertion of the fixed appliance were significantly lower than the values obtained after 6 months of treatment.

## 4. Discussion

The state of oral hygiene and its impact on the intensity of tooth decay, periodontium and general health of orthodontic patients have been a topic of great importance for many years.

Although new appliances, bonding techniques and materials have been developed, it is not yet possible to reduce plaque retention.

The main part of the fixed appliance is located on the vestibular surfaces of the teeth. Traditional fixed appliances create retention areas, difficult for mechanical plaque control. Bracket design, roughness of appliances’ surface, bracket cement excess and elastomeric ligatures affect increased plaque retention. The parts of the appliance make it difficult to clean the teeth with saliva, help to retain food remnants and worsen oral hygiene. Active biofilm disturbs the balance of demineralization and remineralization, leading to the formation of white spots on the enamel especially on the vestibular surfaces of teeth [28]. In this localization, decalcification is not usually observed in patients without orthodontic brackets.

The issue is also very important for oral health. The results of studies assessing the effectiveness of prophylaxis methods aimed at improving oral hygiene during orthodontic treatment are not unequivocal, which makes it difficult to draw conclusions and apply them in orthodontic practice [29].

Many methods have been proposed to improve oral hygiene during orthodontic therapy: preventive measures (instruments and medication), communication methods, frequency and hygiene instructions.

As mechanical plaque control is a challenge for teenage orthodontic patients, who are not aware which areas they overlook during brushing, it is important to instruct patients treated with fixed appliances how to clean all surfaces of the tooth properly [30]. Some researchers indicate that good oral hygiene during orthodontic treatment depends on brushing practice. Patients using normal and interdental toothbrushes with adequate brushing time and frequency are less prone to plaque build-up [31]. The results of studies comparing the effectiveness of various kinds of toothbrushes in the orthodontic patients group do not show unequivocal results [32,33,34,35,36,37,38,39,40]. Mylonopoulou et al. [40] concluded that, as there is no difference in plaque removal between various kinds of toothbrushes, clinicians should rather improve their patients’ awareness of the principles of proper oral hygiene and pay attention to professional prophylaxis and other oral hygiene measures.

Studies on the effectiveness of a particular brushing technique in orthodontic patients are not conclusive [41]. The authors emphasize the need for studies that would compare different brushing techniques and indicate the most appropriate one for patients with fixed appliances.

To ensure efficient biofilm control, tooth brushing alone is not effective enough for most orthodontic patients. Chemical plaque control agents, a proper diet and remineralizing agents are a complement to mechanical plaque control.

Contaldo et al. [42] reported that quantitative changes in plaque in orthodontic patients are observed after just one week after fixation and become more consistent three months after starting treatment. In patients undergoing therapy with fixed appliances, strengthening and regular oral hygiene control are recommended in the first months of treatment.

There are three educational methods useful in achieving optimal oral health during fixed appliances therapy: auditory, visual and kinesthetic [29].

Some authors recommend Motivational Interviewing (MI) which stimulates the patient’s internal motivation and improves compliance [43].

To promote oral health behavior among adolescents with fixed orthodontic appliances, mobile phone apps, e.g., the WhiteTeeth or Brush DJ were developed. These apps work as motivators and reminders, especially in the teenage group [44,45].

Traditional appointments at the dentist’s office can be supplemented with advice given over a phone call. This method allows examining a patient’s compliance with certain rules of proper oral care and provides the motivation.

In our own study, we observed increased plaque formation despite intense individual oral hygiene education, regular controls and motivations.

After insertion of the fixed appliance, we observed deterioration of oral hygiene. The dental plaque indices values in the orthodontically treated group were significantly (*p* < 0.05) higher than in the control group. Significant changes in the distribution of the biofilm were also noticed. This was characterized by the change in the location of the dental plaque. In orthodontic patients, we observed an increase in the amount of supragingival plaque not only on the interproximal but also on the vestibular surface of the teeth. Our observations were in accordance with the results of previous studies. Klukowska et al. [46] observed that plaque was mainly present along the gum line and around the bracket and wire. Mei L. et al. [47] observed that patients with braces have the highest biofilm formation, particularly in the gingival area and areas behind arch wires. Hannig [48] observed early plaque formation on dental and orthodontic materials. The author noticed that, regardless of the material, the pellicle layer on lingual surfaces was much thinner than on buccal surfaces. Erbe et al. [49], evaluating the distribution of biofilm in the regions surrounding orthodontic brackets, found that more plaque was deposited directly in the sensitive supragingival area than in the interdental palatal areas.

The limitation of our study was the lack of dividing patients into groups due to the methods of hygiene used by them.

We conclude that there is a need to develop more effective and modern methods to improve oral hygiene in patients with fixed appliances, especially in areas of the teeth that accumulate more biofilm so that the advice we provide to our orthodontic patients would ensure the fully effective removal of plaque.

Knowing where plaque accumulates is important for orthodontists to implement appropriate prevention strategies. This knowledge about areas that require more attention can help clinicians to implement oral hygiene education appropriate for the patients during fixed appliances therapy. 

## 5. Conclusions

The present study had some limitations. We did not divide patients into groups according to the methods of hygiene used by them.

With this limitation in mind, we can conclude as follows:

1. Fixed appliances caused measurable changes in the distribution of the biofilm, characterized by the change in the location of the plaque.
2. In orthodontic patients we observed an increase in the amount of the supragingival plaque in the interdental area and on the vestibular surface of the teeth.
3. There is a need to change the hygiene protocol and develop modern, more effective methods to improve oral hygiene in patients with fixed appliances, especially in areas of the teeth that accumulate more biofilm, so that the advice we provide to our orthodontic patients would ensure the fully effective removal of plaque.

## Figures and Tables

**Table 1:** Mean values of the Approximal Plaque Index (API) in both groups.

Group	API	*n*	M	SD	V	min	max	Q1	Me	Q3
Orthodontically treated group	API1	60	16.20	13.23	81.7%	0.00	50.00	7.50	13.00	24.00
	API2	60	23.57	16.33	69.3%	0.00	75.00	13.00	18.50	31.50
	API3	60	34.10	17.34	50.8%	0.00	88.00	23.00	33.00	43.00
Control group	API1	84	14.96	11.83	79.08%	0.00	83.00	7.00	13.00	20.00
	API2	84	12.63	13.92	110.2%	0.00	90.00	3.00	10.00	17.00
	API3	84	20.82	20.04	96.3%	0.00	97.00	7.00	13.00	27.00

**Table 2:** Values of the Mann–Whitney U test for Approximal Plaque Index (API).

Comparison	API	U	Z	*p*-Value
Orthodontically treated and control group	API1	2416.0	0.4194	0.6749
	API2	1285.0	5.0237	<0.0001
	API3	1306.5	4.9220	<0.0001

**Table 3:** ANOVA Friedman and Wilcoxon pairwise test values for the Approximal Plaque Index (API).

Group	ANOVA Friedman	*p*-Value	Comparison	Wilcoxon Matched-Pairs Test	*p*-Value
Orthodontically treated group	42.26	<0.0001	API1 API2	3.62	0.0003
			API1 API3	5.64	<0.0001
			API2 API3	4.04	0.0001
Control group	17.51	0.0002	API1 API2	2.22	0.0265
			API1 API3	2.74	0.0061
			API2 API3	4.68	<0.0001

**Table 4:** Descriptive statistics for the Bonded Bracket Index in patients treated with fixed appliances (BBI).

Group	BBI	*n*	M	SD	V	min	max	Q1	Me	Q3
Orthodontically treated group	BBI2	60	1.17	0.96	81.7	0.00	3.60	0.41	1.00	1.85
	BBI3	60	1.51	1.02	68.0	0.00	3.75	0.61	1.31	2.32

**Table 5:** Wilcoxon’s pairwise test values for the Bonded Bracket Index in patients treated with a fixed orthodontic ap-pliance (BBI).

Group	Comparison	Wilcoxon Matched-Pairs Test	*p*-Value
Orthodontically treated group	BBI2 BBI3	2.64	0.0083
